# Supplementary material for: OpenMM 7: Rapid development of high performance algorithms for molecular dynamics
Source: PLoS Comput Biol. 2017 Jul 26;13(7):e1005659. doi: 10.1371/journal.pcbi.1005659 (PMC5549999; doi:10.1371/journal.pcbi.1005659)
Supplement: S1 Source Code — (ZIP) [file pcbi.1005659.s002.zip › OpenMM-7.0.1-Source/docs-source/Lepton User's Manual.doc]

| Documents | |
| --- | --- |
|  |  |
|  | Lepton User’s Manual |
|  | Release 1.3  October 17, 2013 |
|  | Website: SimTK.org/home/lepton |

**Copyright and Permission Notice**

Portions copyright (c) 2009-2013 Stanford University and Peter Eastman.

Contributors: Peter Eastman

Permission is hereby granted, free of charge, to any person obtaining a copy of this document (the "Document"), to deal in the Document without restriction, including without limitation the rights to use, copy, modify, merge, publish, distribute, sublicense, and/or sell copies of the Document, and to permit persons to whom the Document is furnished to do so, subject to the following conditions:

This copyright and permission notice shall be included in all copies or substantial portions of the Document.

THE DOCUMENT IS PROVIDED "AS IS", WITHOUT WARRANTY OF ANY KIND, EXPRESS OR IMPLIED, INCLUDING BUT NOT LIMITED TO THE WARRANTIES OF MERCHANTABILITY, FITNESS FOR A PARTICULAR PURPOSE AND NONINFRINGEMENT. IN NO EVENT SHALL THE AUTHORS, CONTRIBUTORS OR COPYRIGHT HOLDERS BE LIABLE FOR ANY CLAIM, DAMAGES OR OTHER LIABILITY, WHETHER IN AN ACTION OF CONTRACT, TORT OR OTHERWISE, ARISING FROM, OUT OF OR IN CONNECTION WITH THE DOCUMENT OR THE USE OR OTHER DEALINGS IN THE DOCUMENT.

**Acknowledgments**

The development of Lepton is funded by the [Simbios](http://simbios.stanford.edu/) National Center for Biomedical Computing through the National Institutes of Health Roadmap for Medical Research, Grant U54 GM072970. Information on the National Centers can be found at <http://nihroadmap.nih.gov/bioinformatics>.

**Table of Contents**

1	Introduction	5

1.1	Overview	5

1.2	Basic Usage	5

1.3	License	6

2	Using Lepton	8

2.1	Lepton::ParsedExpression	8

2.2	Optimization	8

2.3	Differentiation	10

2.4	Lepton::ExpressionProgram	10

2.5	Lepton::CompiledExpression	11

2.6	Lepton::CustomFunction	12

3	Appendix A	15

# 1 Introduction

## 1.1 Overview

Lepton (short for “lightweight expression parser”) is a C++ library for parsing, evaluating, differentiating, and analyzing mathematical expressions. It takes expressions in the form of text strings, then converts them to an internal representation suitable for evaluation or analysis. Here are some of its major features:

- Support for a large number of standard mathematical functions and operations.
- Support for user defined custom functions.
- A variety of optimizations for automatically simplifying expressions.
- Computing analytic derivatives.
- Representing parsed expressions in two different forms (tree or program) suitable for further analysis or processing.

It is typically used in one of two ways. One case is that you use it to evaluate expressions. This is very easy to do, and is described in the next section.

The other case is that you want to process expressions yourself in some more advanced way. In particular, Lepton was originally created for use in the OpenMM project (<https://simtk.org/home/openmm>). In OpenMM, Lepton is used to parse expressions, differentiate them, and convert them to a representation that is easier to process. OpenMM then transforms that representation into OpenCL or CUDA source code, allowing them to be evaluated on a graphics processor as part of a molecular simulation.

## 1.2 Basic Usage

If all you want to do is evaluate mathematical expressions, this section tells you everything you need to know. You can skip the rest of the manual, except for Appendix A which describes the syntax for expressions and lists the supported functions and operators.

Here is the code to evaluate a simple expression:

```
#include “Lepton.h”
...
double value = Lepton::Parser::parse("sqrt(9)-1").evaluate();
```

To evaluate an expression that involves variables, create a std::map containing values for the variables:

std::map<string, double> variables;

variables["x"] = 2.0;

variables["y"] = 3.0;

double value = Lepton::Parser::parse("x^y").evaluate(variables);

If you plan to evaluate an expression many times with different values for the variables, this is the most efficient way to do it:

Lepton::CompiledExpression expression = Lepton::Parser::parse("2*x+1").createCompiledExpression();

double& x = expression.getVariableReference("x");

// Each time you want to evaluate it, do the following:

x = 3.0;

double value = expression.evaluate();

## 1.3 License

Lepton is Copyright 2009-2013 by Stanford University and Peter Eastman. It is distributed under the following license:

Permission is hereby granted, free of charge, to any person obtaining a copy of this software and associated documentation files (the "Software"), to deal in the Software without restriction, including without limitation the rights to use, copy, modify, merge, publish, distribute, sublicense, and/or sell copies of the Software, and to permit persons to whom the Software is furnished to do so, subject to the following conditions:

The above copyright notice and this permission notice shall be included in all copies or substantial portions of the Software.

THE SOFTWARE IS PROVIDED "AS IS", WITHOUT WARRANTY OF ANY KIND, EXPRESS OR IMPLIED, INCLUDING BUT NOT LIMITED TO THE WARRANTIES OF MERCHANTABILITY, FITNESS FOR A PARTICULAR PURPOSE AND NONINFRINGEMENT. IN NO EVENT SHALL THE AUTHORS, CONTRIBUTORS OR COPYRIGHT HOLDERS BE LIABLE FOR ANY CLAIM, DAMAGES OR OTHER LIABILITY, WHETHER IN AN ACTION OF CONTRACT, TORT OR OTHERWISE, ARISING FROM, OUT OF OR IN CONNECTION WITH THE SOFTWARE OR THE USE OR OTHER DEALINGS IN THE SOFTWARE.

# 2 Using Lepton

Since you are still reading, you presumably want to do something more than just evaluate mathematical expressions. Let’s go through the API in detail and examine how each part of it works.

## 2.1 Lepton::ParsedExpression

The Lepton::ParsedExpression class is the primary internal representation for a mathematical expression. A ParsedExpression is created by passing a string to Lepton::Parser::parse().

A ParsedExpression represents the expression as a tree of Lepton::ExpressionTreeNode objects. Call getRootNode() to get the root of the tree. Each node specifies a list of child nodes (obtained by calling getChildren() on it) and an operation to perform on them (specified by a Lepton::Operation object, obtained by calling getOperation() on it).

A ParsedExpression is evaluated by hierarchically evaluating all nodes in the tree. To evaluate a node, each of its child nodes is first evaluated. Based on the node’s Operation, some calculation is then performed on those values to produce the final value.

Operation is an abstract class. It has many subclasses, each corresponding to a particular value, function, or operator that can appear in an expression. Some examples are Operation::Constant representing a numeric constant, Operation::Multiply representing multiplication, and Operation::Sqrt representing the square root function.

## 2.2 Optimization

Lepton can transform a ParsedExpression in a variety of ways to allow it to be evaluated more quickly. Simply call optimize() on it to create a new, optimized ParsedExpression. Here are some examples:

cout<<Parser::parse("2*3*x").optimize()<<endl;

produces the output

6*(x)

In this case, it has precomputed the subexpression “2*3”.

cout<<Parser::parse("1/(1+x)").optimize()<<endl;

recip(1+(x))

In this case, it has performed two optimizations. The expression “1+x” involves three nodes: 1, x, and the + operator. It has reduced it to only two nodes: x and an “add constant” operation. It also has replaced “1/” (two nodes) with a single “reciprocal” operation.

cout<<Parser::parse("x^(1/2)").optimize()<<endl;

sqrt(x)

It first precomputed “1/2” to get 0.5. It then realized that raising something to the 0.5 power is equivalent to taking the square root. This transformation eliminates a node from the tree, and also takes advantage of the fact that the sqrt() function is often implemented in hardware, and therefore is faster to evaluate than generic exponentiation.

Here is an extreme example of the power of optimization:

cout<<Parser::parse("log(3*cos(x))^(sqrt(4)-2)").optimize()<<endl;

1

It precalculated “sqrt(4)-2”, then recognized that anything raised to the 0’th power is equal to 1.

When calling optimize(), you can provide values for a subset of the variables that appear in the expression. This is useful when symbols have fixed values. Those symbols can then be replaced with the fixed values, and optimizations can be done based on them.

std::map<string, double> constants;

constants["pi"] = M_PI;

cout << Parser::parse("2*pi*x").optimize(constants) << endl;

6.28319*(x)

## 2.3 Differentiation

Lepton can compute the analytic derivatives of expressions. Simply call differentiate() on a ParsedExpression, specifying the name of the variable to differentiate with respect to. For example, the following code evaluates d(x^2^)/dx

cout<<Parser::parse("x^2").differentiate("x")<<endl;

(((2)*((x)^(-1+(2))))*(1))+(((log(x))*((x)^(2)))*(0))

If that answer isn’t quite what you were expecting, trust me that it really is correct! It just has a lot of completely wasted calculation, such as evaluating “log(x)*x^2”, then throwing the result away by multiplying it by zero. It still produces the correct result, but if you plan to evaluate it many times, you should call optimize() to get a simpler expression:

cout<<Parser::parse("x^2").differentiate("x").optimize()<<endl;

2*(x)

To calculate higher order derivatives, just call differentiate() multiple times.

## 2.4 Lepton::ExpressionProgram

In addition to ParsedExpression, Lepton can represent an expression in a second way: Lepton::ExpressionProgram. This consists of a linear sequence of Operations to be executed in order. It is created by calling createProgram() on a ParsedExpression.

An ExpressionProgram is evaluated using a stack. Initially, the stack is empty. For each Operation in the program, any necessary arguments are popped off the stack, the Operation is evaluated, and the result is pushed back onto the stack. When the end of the program is reached, the stack will contain a single value, which is the value of the expression.

This class exists because the program form is more convenient for certain types of analysis. Depending on how you plan to process an expression, you can choose the representation that is most appropriate.

## 2.5 Lepton::CompiledExpression

If you plan to evaluate an expression many times, the most efficient representation is a CompiledExpression. Unlike ParsedExpression and ExpressionProgram, this is an opaque representation. It is used solely for evaluation.

You can create a CompiledExpresion by calling createCompiledExpression() on a ParsedExpression. Instead of using a map to specify variables, CompiledExpression provides its own storage space for variables which you can look up by calling getVariableReference():

CompiledExpression expression = Parser::parse("x+y^2").createCompiledExpression();

double& x = expression.getVariableReference("x");

double& y = expression.getVariableReference("y");

Now you can simply assign the values directly before calling evaluate():

x = 1.0;

y = 2.5;

double result = expression.evaluate();

Because CompiledExpression has mutable internal state, it is *not* thread safe. Never access a single CompiledExpression from two threads at the same time. If you need to write multithreaded code, it is best to give each thread its own copy of the CompiledExpression.

## 2.6 Lepton::CustomFunction

In addition to the standard mathematical functions supported by Lepton, you can define your own functions that may appear in expressions. To do this, you must define a subclass of the abstract class Lepton::CustomFunction. When you call Parser::parse(), give it a std::map with function names as keys and the corresponding CustomFunctions as values:

map<string, CustomFunction*> functions;

WootFunction woot;

functions["woot"] = &woot;

ParsedExpression exp = Parser::parse("7*woot(x)+3", functions);

The object stored in the map is used only during parsing; new objects are created by the parser for any nodes that appear in the expression, and those are deleted automatically when the ParsedExpression is deleted. The expression will therefore remain valid even when the variable “woot” goes out of scope.

A subclass of CustomFunction must implement four methods:

int getNumArguments() const

This method should return the number of arguments expected by the function.

CustomFunction* clone() const

This method should return a new CustomFunction object (allocated on the heap with the “new” operator) that is a clone of this one.

double evaluate(const double* arguments) const

This method should evaluate the function. arguments is an array containing the values of all arguments to the function.

double evaluateDerivative(const double* arguments, const int* derivOrder) const

This method should evaluate the function’s derivatives. arguments is an array containing the values of all arguments to the function. derivOrder is an array specifying the number of times the function has been differentiated with respect to each of its arguments. For example, the array {0, 3} indicates a third derivative with respect to the second argument.

As a simple example, here is a complete CustomFunction subclass that implements the function f(x,y) = 2*x*y:

```
class ExampleFunction : public CustomFunction {
    int getNumArguments() const {
        return 2;
    }
    CustomFunction* clone() const {
        return new ExampleFunction();
    }
    double evaluate(const double* arguments) const {
        return 2.0*arguments[0]*arguments[1];
    }
    double evaluateDerivative(const double* arguments, const int* derivOrder) const {
        if (derivOrder[0] == 1) {
            if (derivOrder[1] == 0)
                return 2.0*arguments[1];
            if (derivOrder[1] == 1)
                return 2.0;
        }
        if (derivOrder[1] == 1 && derivOrder[0] == 0)
            return 2.0*arguments[0];
        return 0.0;
    }
```

};

# 3 Appendix A

Lepton supports the following operators in expressions.

| + | Add |
| --- | --- |
| - | Subtract |
| * | Multiply |
| / | Divide |
| ^ | Power |

The following mathematical functions are built in. Others may be added using the CustomFunction class.

| sqrt | Square root |
| --- | --- |
| exp | Exponential |
| log | Natural logarithm |
| sin | Sine (angle in radians) |
| cos | Cosine (angle in radians) |
| sec | Secant (angle in radians) |
| csc | Cosecant (angle in radians) |
| tan | Tangent (angle in radians) |
| cot | Cotangent (angle in radians) |
| asin | Inverse sine (in radians) |
| acos | Inverse cosine (in radians) |
| atan | Inverse tangent (in radians) |
| sinh | Hyperbolic sine |
| cosh | Hyperbolic cosine |
| tanh | Hyperbolic tangent |
| erf | Error function |
| erfc | Complementary error function |
| abs | Absolute value |
| min | Minimum of two values |
| max | Maximum of two values |
| delta | delta(x) = 1 if x=0, 0 otherwise |
| step | step(x) = 0 if x<0, 1 if x>=0 |
| floor | Floor |
| ceil | Ceiling |
| select | select(x,y,z) = z if x=0, y otherwise |

Numbers may be given in either decimal or exponential form. All of the following are valid numbers: 5, -3.1, 1e6, and 3.12e-2.

An expression may be followed by definitions for intermediate values that appear in the expression. A semicolon “;” is used as a delimiter between value definitions. For example, the expression

a^2+a*b+b^2; a=a1+a2; b=b1+b2

is exactly equivalent to

(a1+a2)^2+(a1+a2)*(b1+b2)+(b1+b2)^2

The definition of an intermediate value may itself involve other intermediate values. All uses of a value must appear *before* that value’s definition.
